# Supplementary material for: Mapping the evolution of fertility support policies in China: A content and instrumental analysis
Source: PLoS One. 2025 Oct 9;20(10):e0332137. doi: 10.1371/journal.pone.0332137 (PMC12510515; doi:10.1371/journal.pone.0332137)
Supplement: S1 Appendix — (ZIP) [file pone.0332137.s001.zip › S1 Appendix. 226 original policy documents/111-人力资源和社会保障部关于进一步促进宁夏人力资源和社会保障事业发展的意见(FBM-CLI-4-114513).doc]

人力资源和社会保障部关于进一步促进宁夏人力资源和社会保障事业发展的意见

**发布部门：** [人力资源和社会保障部](https://www.pkulaw.com/law/chl?Aggs.IssueDepartment=60236) [机构沿革](javascript:void(0))

**发文字号：**人社部发〔2008〕105号

**发布日期：**2008.11.30

**实施日期：**2008.11.30

**时效性：** [现行有效](https://www.pkulaw.com/law/chl?Aggs.TimelinessDic=01)

**效力级别：** [部门规范性文件](https://www.pkulaw.com/law/chl?Aggs.EffectivenessDic=XE0302)

**法规类别：** [社会福利与社会保障](https://www.pkulaw.com/law/chl?Aggs.Category=01505)

人力资源和社会保障部关于进一步促进宁夏人力资源和社会保障事业发展的意见
（人社部发[2008]105号）

各省、自治区、直辖市人事厅(局)、劳动保障厅 (局)，新疆生产建设兵团人事局、劳动保障局：
　　为全面贯彻落实党的十七大精神，深入学习实践科学发展观，根据《[国务院关于进一步促进宁夏经济社会发展的若干意见](https://www.pkulaw.com/chl/e7c82f24fe07e544bdfb.html)》　(同发[2008] 29号)的要求，为进一步促进宁夏人力资源和社会保障事业的发展，现提出以下意见：
　　**一、**充分认识促进宁夏人力资源和社会保障事业发展的重要意义
　　宁夏回族自治区是我国重要的少数民族自治地区、革命老区和集中连片贫困地区，在推进西部大开发和区域协调发展战略中占有重要地位。近年来，宁夏各级人力资源和社会保障部门以改善民生为出发点和落脚点，大力实施人才强区战略，人力资源和社会保障事业取得积极进展。当前是宁夏加速推进工业化、城镇化和农业现代化，为全面建设小康社会奠定基础的关键时期，宁夏人力资源和社会保障事业既面临难得的历史机遇，也面临着一些突出困难和问题：就业总量供大于求，就业形势比较严峻；劳动权益社会保障体系尚不健全，基金支撑能力相对薄弱；人才总量不足，高层次人才严重缺乏；工资收入分配制度有待进一步完善；劳动关系日趋复杂，矛盾突出；人力资源和社会保障基础能力建设亟待加强。必须从战略和全局的高度出发，鼓励和支持宁夏人力资源和社会保障部门在新的起点上，抓住机遇，克服困难，解放思想，采取更加有力的措施，不断推进宁夏人力资源和社会保障事业的发展。

　　**二、**实施积极的就业政策，千方百计扩大就业
　　(一)继续扩大就业规模和优化就业结构。支持宁夏继续实施积极的就业政策，坚持统筹城乡就业，努力扩大就业规模，改善就业结构。大力发展旅游业等第三产业，提高第三产业的就业比重。鼓励和支持有利于扩大就业的劳动密集型行业、中小企业、非公有制经济更好发展，更多吸纳就业。以促进创业带动就业，完善鼓励城乡劳动者创业的各项政策，优化创业环境，搭建创业服务平台，培育创业服务队伍，在2-3个城市开展创建创业型城市工作。
　　(二)加大对就业困难人员的就业援助力度。支持宁夏落实各项扶持政策，开展就业援助，开发公益性岗位，重点帮扶零就业家庭和就业困难人员实现就业。帮助其他困难人员多渠道实现就业。进一步加大中央就业补助资金对宁夏的转移支付力度，完善社会保险、职业培训、职业技能鉴定、职业介绍和公益性岗位补贴办法，扩大小额担保贷款基金总量和扶持范围，完善小额担保信用体系，推动创建信用社区试点工作。
　　(三)强化高校毕业生就业服务。加大高校毕业生就业指导力度，指导公共就业服务机构为高校毕业生提供免费就业服务。鼓励高校毕业生到宁夏基层和边远地区就业，推动落实到西部和艰苦边远地区基层就业毕业生的助学贷款代偿、报考研究生优先录取、户档迁转、高定工资标准等优惠政策。做好“三支一扶”计划实施工作，重点向宁夏南部贫困山区倾斜。做好项目期满人员的报录(聘)和就业服务工作。全面建立高校毕业生就业见习制度和就业见习基地。充分运用现行政策措施和服务手段，着力解决登记失业的高校毕业生就业。
　　(四)加强就业和创业培训。继续指导和支持宁夏实施农村劳动力技能就业计划和创业培训，加大培训补助力度，不断提升培训质量。有针对性地开展职业技能培训和就业服务，继续组织实施好“城乡贫困和零就业家庭培训就业援助工程”。在职业教育基础能力建设项目、技能培训试点示范项目、培训师资能力提升等方面给予宁夏倾斜，支持宁夏以技工学校、技师学院为基础建立一批公共实训基地。
　　(五)加大农业富余劳动力转移培训力度。加强对农业富余劳动力的职业技能培训，支持宁夏结合实际实施农村劳动力技能就业计划。积极支持宁夏开展劳务输出示范县创建活动，加强劳务协作和劳务基地建设，巩固和拓展劳务市场。精心培育具有地方特色和竞争优势的劳务输出品牌，通过劳务品牌占领市场，增加收益。
　　(六)大力推进素质就业工程。加强职业技能培训和鉴定服务体系建设，完善职业资格证书制度，加强职业技能鉴定工作，开展企业技能人才评价试点工作，强化高技能人才评价工作。继续实施新技师培养带动计划。加强职业培训教师队伍建设，每年安排部分师资到东部和沿海发达地区学习提高，建立起适应职业技能培训和技工院校发展的师资队伍。
　　(七)加强人力资源市场建设，完善公共就业服务体系。按照制度化、专业化、社会化要求，加快建立统一规范的人力资源市场。指导和支持宁夏建立健全公共就业服务体系，实现地市和县级公共就业服务机构信息联网，重点加强乡镇、街道(社区)基层劳动保障服务体系和工作机制建设。支持社会力量举办的人力资源服务机构的发展。推动人力资源服务机构的跨区域合作。进一步完善服务功能，强化服务手段和服务效能。

　　**三、**加快完善社会保障体系
　　(一)改革和完善各项社会保险制度。支持宁夏进一步改革和完善现有各项社会保险制度，逐步建立统筹城乡的社会保障体系。加快建立完善农民工和被征地农民的社会保险制度。不断扩大社会保险覆盖范围，加强社会保险费征缴，逐步提高社会保险统筹层次，增强社会保险基金统筹调剂能力。帮助宁夏解决社会保障历史遗留问题。
　　(二)强化社会保险基金管理和监督。指导宁夏建立健全各级社会保险基金监督机构，完善社会保险经办机构内控机制和社会保险基金信息定期披露制度，建立行政监督、专门监督、社会监督、内部控制相结合的监督体系。健全社会保险基金预决算制度，加大各级财政对社会保障资金的支持力度，建立规范的社会保障资金筹集和支出制度，确保各项基金安全完整。
　　(三)建立健全社会化管理服务体系。加强社会保险经办能力建设，整合社会保险经办机构和工作职能，加快“五险”统一征缴步伐，实现社会保险业务集中办理。加强乡镇、街道(社区)劳动保障平台建设，加强基础管理，整合服务资源，拓展服务内容。加快公共老年服务设施、服务网络建设和社会保险服务中心建设，在统一规划下支持和鼓励有条件的地方兴建退休人员公寓、工伤人员康复中心，不断提高社区管理服务水平。
　　(四)继续改革和完善养老保险制度。构建覆盖城乡居民的养老保险制度，逐步实现由城镇为主向城乡统筹、由城镇职工为主向城乡居民的重大转变，努力实现养老保险制度的全覆盖。进一步完善省级统筹制度。积极稳妥推进做实养老保险个人账户试点。改革企业养老金计发办法，建立多工作、多缴费、多得养老金的激励约束机制。逐步提高企业离退休人员养老金水平。鼓励有条件的企业建立企业年金。积极推进机关、事业单位的养老保险制度改革。加快推进被征地农民养老保障工作，建立农民工养老保险制度，支持宁夏探索解决城镇无收入困难老年居民养老保障的机制和办法。加大各级财政对建立新型农村社会养老保险制度的投入，支持宁夏开展个人缴费；集体补助、政府补贴相结合的新型农村养老保险试点。
　　(五)扩大基本医疗保险覆盖范围。不断完善城镇职工基本医疗保险制度，逐步扩大基本医疗保险覆盖范围。加快城镇居民基本医疗保险试点工作，将宁夏所有城市(地区)纳入试点范围，中央财政对居民参保缴费给予适当补助。完善生育保险制度，进一步扩大生育保险覆盖范围。提高医疗、生育保险统筹层次。
　　(六)完善失业保险制度。充分发挥失业保险促进就业的作用，加大对就业再就业的支持力度，实现失业保险与促进就业的联动。以事业单位和非公有制经济组织及其从业人员为重点，扩大失业保险覆盖范围。适时提高失业保险金水平，确保失业人员基本生活。支持宁夏建立失业保险省级统筹制度。加强失业预防工作，建立失业预警和失业动态重点监测报告制度。　　．
　　(七)完善工伤保险制度。继续扩大工伤保险覆盖范围，进一步推进高风险企业参加工伤保险，扎实推进农民工参加工伤保险，全力推进事业单位参加工伤保险。加快工伤保险制度建设，积极探索工伤补偿与工伤预防、工伤康复相结合的有效途径。支持宁夏建立工伤保险省级统筹制度。

　　**四、**实施人才强区战略，加强人才队伍建设
　　(一)实施高层次和急需紧缺人才培养工程。根据宁夏经济社会发展需要，加快培养一批创新能力较强的高层次专业技术人才和高技能人才。在享受国务院特殊津贴人选选拔、　“新世纪百千万人才工程”　国家级人选培养等方面向宁夏适当倾斜。在博士后科研流动(工作)站设站审批、国家资助博士后研究人员日常经费和科研经费等方面给予重点支持。支持宁夏开展创建博士后科研创业基地试点工作，促进博士后科研成果转化。协调国内知名高校、科研院所和大型企业，帮助宁夏培养急需紧缺人才。支持宁夏编制和实施人才队伍建设中长期规划。
　　(二)实施专业技术人员素质提升工程。围绕国家专业技术人员知识更新工程，针对宁夏经济社会发展重点领域、特色优势产业、重点学科，适当增加高级研修班班次，支持宁夏完善专业技术人员继续教育制度，建立继续教育基地。按照国务院要求研究开展宁夏少数民族科技骨干特殊培养工作。探索建立专业技术人员继续教育对口援宁机制，协调东部沿海和经济发达地区每年面向宁夏开展继续教育对口支援。支持宁夏加大专业技术人员出国(境)研修力度。
　　(三)实施农村实用人才开发工程。支持宁夏实施“412农村实用人才带头人培训工程”。为宁夏培养设施农业、生态农业、特色产业、农产品深加工等领域农村实用人才提供支持。为基层科技服务人员、农村实用人才到农业发达地区进修创造条件，带动大批农村实用人才为农业和 农村服务。
　　(四)积极引进高层次和紧缺急需人才。支持宁夏引进各方面的高层次和高技能急需紧缺人才，大力加强柔性引进。支持宁夏对引进并在宁夏服务累计半年以上、取得显著经济效益和社会效益的两院院士及其他高层次、高技能人才，给予专项支持。长三角地区有关省市要积极落实与宁夏签订的人才开发合作交流协议。对宁夏从区外、国外引进的高层次、高技能急需紧缺人才，简化程序，特事特办、急事急办。
　　(五)加大引进国外智力的支持力度。按照国务院将宁夏建成“六大基地、六个示范区和一个目的地”的战略定位，加大对宁夏引进国外技术、管理人才项目和出国　(境)培训项目的支持力度，特别是重点项目、东欧和独联体专项、软件与集成电路专项。支持高校学科建设和科技带头人培养，聘请外教开展讲学和科研合作。
　　(六)建立国家级专家服务基地。支持宁夏建立专家服务基地，适时组织国内外专家采取“专家西部服务行”等多种形式，提供咨询服务，开展技术攻关，帮助宁夏解决科研和生产中的重大技术难题。支持和帮助宁夏做好“专家西部服务行”活动成果的转化和运用。
　　(七)鼓励和支持海外高层次留学人才到宁夏工作和创业。加大对宁夏留学人员回国工作的支持力度，重点支持宁夏引进高新技术产业领域的高层次留学人才。支持宁夏建设煤化工、农产品深加工2个留学人员创业园，鼓励和支持国内具有一定优势的留学人员创业园与宁夏留学人员创业园开展项目合作。帮助宁夏建立海外留学人员联系和沟通机制，组织海外留学人员和留学回国专家赴宁夏开展为国服务活动。

　　**五、**进一步做好工资收入分配工作
　　(一)严格落实工资福利政策，切实保障宁夏机关事业单位工作人员的工资福利待遇。认真贯彻落实国家关于机关事业单位工作人员工资福利待遇的各项政策规定，确保兑现到位。国家在不断完善艰苦边远地区津贴制度时，统筹研究宁夏的艰苦边远地区津贴问题。结合规范津贴补贴和建立地区附加津贴制度，加强调查研究和工作指导。按国家有关规定，对在宁夏事业单位工作的高层次人才建立激励机制，吸引和留住高层次人才。
　　(二)进一步规范企业工资分配秩序。继续推进企业工资决定机制的转变，着力建立工资集体协商制度。健全最低工资制度，完善工资指导线、劳动力市场工资指导价位和人工成本信息指导等宏观指导制度。完善企业工资支付保障机制，建立健全工资支付监控制度和建筑业、餐饮、服务等重点行业的工资保证金制度。

　　**六、**积极构建和谐劳动关系，加强劳动维权能力建设
　　(一)推进实施劳动合同制度和集体合同制度。认真贯彻落实《[劳动合同法](https://www.pkulaw.com/chl/fd21a70343e50c18bdfb.html)》和《[劳动合同法实施条例](https://www.pkulaw.com/chl/aae289ed71e4880abdfb.html)》，督促指导各类用人单位与全体劳动者签订劳动合同。以非公有制企业为重点，进一步推进集体协商和集体合同制度，扩大集体合同覆盖面。加强协调劳动关系三方机制建设，推进和谐劳动关系创建活动。
　　(二)加强劳动争议处理能力建设。建立注重预防、鼓励、调解、加强仲裁诉讼救济的劳动争议处理机制。推进劳动争议仲裁机构实体化建设，加强劳动人事争议调解仲裁员队伍建设，建立健全企业劳动争议调解组织，大力推进区域性、行业性劳动调解组织建设和市、县(区)劳动争议仲裁实体化办案机构建设，不断完善劳动人事争议调解仲裁工作制度。
　　(三)加强劳动保障监察执法能力建设。建立健全用人单位劳动保障守法诚信制度，完善违反劳动保障法律法规综合治理机制。开展劳动保障监察“网格化”和“网络化”管理，将宁夏纳入全国“两网化”试点范围。协助宁夏解决劳动保障监察执法及“两网化”设备、资金短缺等问题，推动宁夏劳动保障监察执法能力建设。

　　**七、**深入实施[公务员法](https://www.pkulaw.com/chl/823ca97b7f55d18cbdfb.html)，加强宁夏公务员队伍建设
　　(一)认真组织实施《[公务员录用规定(试行)](https://www.pkulaw.com/chl/10a868c7f480baa6bdfb.html)》。坚持“凡进必考”，实行依法考录、科学考录、公平考录，加强对宁夏公务员考录工作的指导，并给予适当政策倾斜。在宁夏公务员考录笔试、面试工作中给予支持，加强公务员统计工作，加快公务员管理信息系统建设。
　　(二)大力开展公务员培训工作。按照中央大规模培训干部的要求，对宁夏公务员培训实行适当政策倾斜，形成多层次、多渠道、分类别的公务员培训工作格局。支持宁夏开展网络培训，利用现代化培训手段，对公务员进行全员培训。继续开展东西部公务员对口培训，在培训班次的设置和名额上进一步向宁夏倾斜。鼓励宁夏公务员特别是年轻公务员，本着工作需要、学用一致的原则，参加有关学历学位教育。

　　**八、**加强对宁夏人力资源社会保障工作调研和指导
　　要围绕宁夏人力资源和社会保障事业发展亟待解决的重大问题，加强调查研究，制定和完善相关政策，有针对性地进行工作指导，将各项政策措施落到实处。有关省(区、市)人力资源和社会保障部门要利用本地优势资源加大对宁夏的支持力度，形成制度化、规范化的援助机制。宁夏各级人力资源和社会保障部门要进一步解放思想，坚持自我发展与国家的支持相结合，抓住历史机遇，提高创新与发展能力，努力开创宁夏人力资源和社会保障事业发展的新局面。

　　二00八年十一月三十日

© 北大法宝：（[www.pkulaw.com](https://www.pkulaw.com/)) 专业提供法律信息、法学知识和法律软件领域各类解决方案。北大法宝为您提供丰富的参考资料，正式引用法规条文时请与标准文本核对。
欢迎查看所有[产品和服务](https://www.pkulaw.com/helps/2.html)。法宝快讯：[如何快速找到您需要的检索结果？](https://www.pkulaw.com/helps/70.html)[法宝](https://www.pkulaw.com/helps/69.html)V6有何新特色？

扫描二维码阅读原文

**原文链接：**<https://www.pkulaw.com/chl/8ec2f7b3cd409f5dbdfb.html>
